# Understanding alcohol as an element of ‘care practices’ in adult White British women’s everyday personal relationships: a qualitative study

**DOI:** 10.1186/s12905-018-0629-6

**Published:** 2018-09-05

**Authors:** Katherine Jackson, Tracy Finch, Eileen Kaner, Janice McLaughlin

**Affiliations:** 1 Institute of Health and Society, Newcastle University, The Baddiley-Clark Building, Richardson Road, Newcastle upon Tyne, NE2 4AX UK; 2 Department of Sociology, University of Newcastle, Claremont Bridge Building, Newcastle upon Tyne, NE1 7RU UK

**Keywords:** Care practices, Women, Relationships, Alcohol, Intervention

## Abstract

**Background:**

In the last thirty years there has been a rise in harmful alcohol use amongst White British women. Approaches to alcohol harm reduction typically position drinking as an individual behaviour, with an emphasis on people to make changes to and by themselves. Moving away from an individual approach, this paper works with a relational framework to develop understanding of non-dependent women’s drinking in the context of their everyday lives. It draws on Feminist Ethics of Care theory, to consider the importance of care in women’s lives and alcohol as an element of their ‘practices of care’ in different relationships.

**Methods:**

The study adopted an interpretive approach and drew on feminist principles of practice. Qualitative one-to-one face-to-face interviews were undertaken with twenty-six White women living in the North East of England. Participants were aged between 24 and 67 years. Thematic analysis of the data was carried out.

**Results:**

Participants’ relationships came through the analysis as central to understanding the way alcohol did and not feature in care practices. In couple relationships drinking offered a way of doing ‘care’ together, yet when it was used too often it no longer became appropriate as a form of care. In non-family relationships alcohol enabled care giving and receiving, while disguising that care was being received. In relationships with mothers the use of alcohol was relatively absent in the care practices described. Participants’ relationship to alcohol as a form of care of self, particularly when drinking alone, was closely related to their roles and responsibilities to others.

**Conclusions:**

Overall the data suggests that interventions targeting women’s drinking should start from a position that women are relational. Moreover that when care by others is lacking or unavailable, alcohol can increasingly be introduced into care practices, and the reproduction of these practices may be leading to an increase in heavy drinking. By seeing alcohol use in the context of wider familial and non-familial relationships, this work has important implications for future interventions.

**Electronic supplementary material:**

The online version of this article (10.1186/s12905-018-0629-6) contains supplementary material, which is available to authorized users.

## Background

In Britain, over the last 30 years, women’s alcohol use has developed as a public health concern [1, 2]. While the media has focused predominantly on young women’s public drinking, there has been an increase in the frequency of women’s drinking overall, and a rise in heavy drinking in most social groups of women [2]. Data from the 2014 ‘Opinions and Lifestyle Survey’ reports that half of all British women had drank alcohol in the last week and a third of these had consumed more than the recommended amount on at least one occasion in the past 7 days [3]. Drinking trends in Britain reflect a recent systematic review of international evidence that suggests, that while women still drink less than men, gaps in men and women’s alcohol use and related harms are closing [4–6]. In Britain most minority ethnic groups have more abstainers and lower rates of harmful drinking, in comparison to other ethnic and minority groups [7]. Alcohol statistics show that White British women are the least likely ethnic group of women to be abstainers, and the most likely to drink above the recommended safe alcohol guidelines in a week [8]. Although alcohol use in ethnic minority groups of women in Britain warrants further research, as alcohol consumption may be increasing in some ethnic groups of women [7], the focus of this article is White British women who are currently most at risk of health and social harms from their alcohol consumption. Drinking at any level can increase the risk of up to 60 diseases including cancers, cardiovascular and liver diseases, as well as an increase in the risk of injury; a pattern which for most diseases increases with levels of consumption, with women particularly vulnerable at lower levels of consumption [6]. While not all White British women are involved in drinking practices that are considered risky to their health, the increasing pattern of consumption amongst some needs to be engaged through sensitive and appropriate evidence-based intervention approaches.

In this paper, drawing on data from a National Institute for Health Research funded study of women’s alcohol use and stress, the aim is to develop understanding of how alcohol use is embedded within women’s wider practices. From analysis of the data, it became clear that the relational aspects of the participants’ lives, were very important to patterns of alcohol use. To explore this, the paper draws from a relational framework [9, 10], focused on the interdependencies of care that are a central feature in women’s lives. In doing so it considers how alcohol may have become incorporated – at times problematically – into care practices. In the conclusion there is a consideration of the inferences that can be drawn from this about approaches to developing interventions to support a reduction in women’s alcohol use. The section below briefly summarises the approach to care.

### Care practices and feminist ethics of care theory

In Britain, as in other Western countries, care is still widely considered as an attitude or disposition [9, 10]. Expectations of care are gendered, with women, families and households perceived as the main source of support and care both day-to-day and at times of crisis [11]. Women normally take on greater responsibilities for the care of partners, and other family members. In particular, mothers are still regarded as the primary carers of children, more able and willing to take the lead in their care, over and above themselves [12]. Overall, moral obligations and expectations of some gender roles, leave women over represented in care giving and without opportunities for receiving care in return. In the last century there has been a rapid period of social change and in 2013 almost three quarters of British women (67%) of working age (16–64 years) were in paid work outside the home [13]. However, empirical studies of families show that despite some changes towards greater equality, overall, gendered inequalities in relation to: domestic work, childcare and care for other family members persist [14, 15].

Feminist Ethics of Care Theory is one approach within feminist research that tries to understand why women continue to carry the responsibility to care in the context of other social changes. To be dependent, or in need of care, in contemporary Britain is often seen as a weakness [9], viewed in opposition to the values of autonomy and independence [16, 17]. In contrast, from a Feminist Ethics of Care approach, care is viewed as necessary and ‘central to daily life’ [10, 18]. It emphasises that all men and women are interdependent, and that these interdependencies generate care practices, which are necessary preconditions for everyday wellbeing and agency [19]. Care is difficult to define but this paper adopts Fisher and Tronto’s [20] broad definition of care that encapsulates the idea that care helps to *maintain*, *continue* and *repair*. They state ‘On the most general level, we suggest that caring be viewed as a species activity that includes everything that we do to maintain, continue and repair our “world” so that we can live in it as well as possible. That world includes our bodies, ourselves, and our environment, all of which we seek to interweave in a complex, life-sustaining web.’

Central to the Feminist Ethics of Care argument is that care is not sentimentalised as a disposition or emotion. Instead it is viewed as a social practice [9]. Social practice approaches view everyday human actions and behaviour, in this case care, as the observable performance of a number of interacting elements [21]. Shove et al. [21] describe three elements that make up social practices: 1.) materials: objects, infrastructures, hardware and the body itself; 2.) competencies: multiple forms of understanding and practical knowledge; 3.) meanings: the social and symbolic significance of participation at any one moment. The value of thinking of care as a form of social practice is it enables us to move away from thinking of care as a natural female activity. It reveals that whether care is offered, and what is considered good and bad care, depends on the context [10, 18]. It makes more visible that the inequalities that exist in terms of who gives and receives care [9], and the boundaries around where it takes place, can leave people without care.

Like other social practices, care practices may also involve material objects as giving meaning to care [9]. Mol, Moser and Pols [18] have described how objects come to be incorporated into care practices and, in some cases, come to be seen as good care, while in others they do not. It is the use of alcohol as part of care practices that is the focus of this article.

The emerging literature on women’s everyday alcohol use suggests that one meaning given to drinking is as a social activity, which enhances relationships and provides opportunities for social interactions [22–26]. The majority of these studies focus on alcohol use in young women’s (aged 16–25 years) friendship relationships. For these women, alcohol is seen to enable bonding, facilitate interactions [23, 24] and can lead to emotional support or sharing problems [25, 26]. There are still few qualitative studies of adult British women’s everyday alcohol use. But in their recent study of Scottish women’s everyday drinking in midlife Emslie et al. [27] describe how their female participants (aged 30–50 years) who were settled in relationships, or had caring responsibilities (they exclude participants who had freedom to go out drinking after work), used alcohol as ‘time out - from responsibilities. ‘Time out’ offered an opportunity to spend time with their partner, or to socialise with female friends - a break from paid work or childcare. For the women with young children in their sample (under 5 years) the authors describe that alcohol provided a ‘transformative space’, a way for the women to maintain a sense of their adult identity or to return to their younger and freer selves. This supports and extends other work about women’s drinking in adulthood, which views alcohol as a social activity rather than merely a health behaviour, enabling breaks from roles and responsibilities, across a range of relationships [28–30].

This work on the role alcohol plays in social interactions and as a form of ‘time out’ is valuable; what remains a gap in the literature is examining how alcohol use is given different meanings in care practices in family and non-family relationships. Particularly how normative expectations of care in different relationships, and gaps in care in these relationships, might lead to reoccurring heavy alcohol use, problematically being used as a version of self-care.

## Methods

This research was conducted as part of the first author’s (KJ) National Institute for Health Research doctoral fellowship awarded between 2010 and 2016. The data reported in this article was collected during 2014 and 2015. The overall aim of the project was to use qualitative methods to explore women’s drinking in relation to the stress they described in their everyday lives, and make recommendations for intervention approaches to alcohol harm reduction from the findings. This paper reports one of the major conceptual themes from the research.

### Approach

The project adopted an interpretive approach drawing on feminist principles of practice [31]. In an interpretivist approach meaning, context and interpretation are important, and over simplification of phenomena to causal mechanisms is avoided by trying to get closer to the ‘individual’s point of view’ [32]. Moreover from this approach, the methods aim to gather data that is co-created in the interaction between the interviewer and the interviewee [31]. A principle of feminist methods as described by Ackerely and True [33] is to do research ‘of value to women and that could result in actions that are beneficial to women.’ This was the overall ambition of the study.

### Context and sampling

The population for the study was women who live in Tyne and Wear and Northumberland, in the North East of England. White British women in the North East of England have some of the highest levels of heavy drinking in the country [3], and the most alcohol related hospital admissions for females [34]. A women only sample was adopted to focus on social and economic differences amongst this group. This intersectional approach acknowledges that women will have different access to power and ‘resources’, which will play out in different ways, and impact on the way that they experience their health [35]. The initial study inclusion criteria was adult women of working age who are legally allowed to purchase alcohol and who were drinking at different levels. A decision was made to exclude women who were currently in treatment for alcohol use disorders. This criteria was developed to fit the aim of the project, which was to understand how drinking sits within women’s lives, so the study could make inferences about interventions that could support a reduction in women’s alcohol use.

Recruitment for the study took place in three phases between June 2014 and June 2015. The sampling techniques were the three qualitative sampling approaches [36]: convenience sampling, stratified purposeful sampling, and the method of trying to identify information rich cases. In the first wave of convenience sampling, a diverse group of women in terms of age and socio-economic circumstance were recruited through dissemination of information about the project through relevant groups and community contacts. In the second wave, further convenience sampling took place through social media and word of mouth, this method recruited a range of participants in terms of circumstances, although fewer from the most deprived socio-economic circumstances and women who were mothers of young children. Thus, the third wave used stratified purposeful sampling to recruit mothers from more deprived areas through a local school and children’s centre. At the end of this third wave an effort was made to recruit information rich cases who had experiences of heavy drinking.

In total the female researcher (KJ), a non-clinician with a social science background, carried out all 26 face-to-face, one-off interviews in participants’ homes, workplaces and community venues. They each lasted around 1 hour and were audio-recorded using a digital recording device.

### Method

The method of semi-structured interviewing was adopted [37]. Key themes structured the interview schedule to focus the interview on exploring women’s alcohol use in relation to stress, while the specific questions were left open to allow participants to expand on the meanings and values associated with the practices they explored. An additional text file shows this interview schedule in more detail (see Additional file 1). It was thought participants would be more likely to discuss potentially sensitive issues on a one-to-one basis, and face-to-face, rather than in a group or virtual situation. Individual interviews also allowed for an opportunity to focus on particular issues introduced by individual participants, and to keep the conversation focused on the interview topic. This ensured rich data was captured about these experiences. Most interviews opened with the question ‘Please could you tell me a bit about your everyday life at the moment’. As the interview process progressed and analysis was being carried out alongside the data collection, new categories emerging from analysis were incorporated into the interviews. The researcher made written fieldnotes after each interview. Interviews were transcribed verbatim for analysis.

### Analysis

The data was analysed using techniques of inductive thematic analysis [38]. Summaries of individual interviews were made as soon as possible after each interview. Early analysis involved familiarisation with the data through multiple listening and re-reading of the transcripts and summaries, and abductive coding. At the end of the first wave of data collection, codes were compared and contrasted across transcripts, these codes were grouped and collapsed under themes, and these themes and codes formed the basis for the initial coding framework. Throughout wave one and two of data collection analysis continued in parallel. New codes and themes were added to the coding framework while others became less significant. The first author coded all transcripts, and 25% of transcripts were also coded by an additional team member.

The main phase of analysis for the argument presented in this paper occurred after data collection, it was at this stage that ‘care’ was identified as a framing concept across the data set. Data related to care was extracted by the first author through abductive coding; this involved identifying semantic ‘surface level’ and latent ‘interpretive’ themes. Feminist Ethics of Care Theory and other feminist approaches to care were applied to the data and have been used to form the argument. It was agreed that data sufficiency was met for the themes and ideas presented here [39].

### Ethical issues

The topic of alcohol use and stress was regarded as potentially sensitive. Some participants did become upset during the interviews and this was managed by the researcher on an individual basis, responding to individual need. Every effort has been made to preserve the anonymity of participants; therefore their names, and other identifiable information about them have been changed. In this paper pseudonyms are used when referring to participants; their real names are never given.

## Results

### Sample characteristics

The final sample are presented in Table 1. Participants included 26 adult White women aged 24–67 years. Twenty-five were White British and one was White Irish, all lived in the North East of England. Deprivation scores from 1 to 10 (with 1 being most deprived and 10 being least deprived) were assigned to participants’ home postcodes [40] as a proxy indicator of their socio-economic status. These deprivation scores indicated participants were from a range of socio-economic backgrounds and the scores have been broken down into most deprived and least deprived as shown in Table 1. There was also a variety of household compositions and relationship statuses. Nineteen participants were in a relationship and seven were single. Fifteen women lived with their children, of the five women who had adult children, four of them had adult children living at home. Sixteen of the women were in paid employment, eight were not in paid employment (seven were caring for their family, one was looking for work) and two were full-time postgraduate students. The participants were not asked to self-identify their sexuality however, twenty-two women spoke about being or having been in mixed-sex couple relationship, two said they were or had been in same sex relationships and two participants did not discuss their sexuality. Participants’ alcohol use was explored qualitatively and all but one were current drinkers.

**Table 1:** Aggregated demographic characteristics of study participants

Participant characteristic	Characteristic Value	Count
Socio-economic status based on Index of multiple deprivation (IMD) score	Most deprived (IMD score 1–5)	13
	Least deprived (IMD score 6–10)	13
Ethnicity	White British	25
	White Irish	1
Age group	20–29 years	6
	30–39 years	9
	40–49 years	8
	50–59 years	2
	60+ years	1
Employment status	In paid employment	16
	Not in paid employment	8
	Full-time student	2
Parenting status	Non-mother	10
	Mother of dependent children only	11
	Mother of adult children only	3
	Mother of dependent and adult children	2
Relationship status and household composition	Married or in a couple relationship and living together with dependent and/or adult children	11
	Married or in a couple relationship and living together without children	7
	In a couple relationship lives alone with children	1
	Single lives alone with dependent and /or adult children	3
	Single lives alone	4

### Overview of themes

The women’s interdependencies and relationally were evident in their full narratives of alcohol use in their everyday lives. A summary of the sub-themes that were identified during the overall analysis are presented in Table 2 below.

**Table 2:** Overall analysis themes and sub-themes

Broad topic area/ theme	Sub-themes
Everyday pressures and demands- stress	• Not feeling cared for • Lack of ‘time for me’ • Unmet expectations of care • Negotiating informal/unpaid care practices and paid work
Social meanings of alcohol use	• Time out - ‘Time for me’/Adult time • Time out - Not thinking/temporarily forget • Social interaction/Social support • Empowering
Regulation and control of alcohol use	• Performing roles and responsibilities • Not drinking alone/at home • Embodied ‘cut off limit’ • Not wanting to waste a day • Alcoholic personality

Care giving and receiving was important in a range of the women’s personal relationships, and the focus of this article is alcohol as part of ‘care practices’ in these relationships which was identified as a framing concept across the data. Sub-themes related to ‘care practices’ are highlighted in the text and the sub-themes are summarised in Table 3 below. The discussion of the data is also separated into the relationship contexts of; being alone, couple relationships, non-family relationships (this term is used here to include friends, neighbours, and colleagues), and relationships with mothers.

**Table 3:** Sub-themes for the framing concept ‘Care practices’

Framing concept	Sub-themes
‘Care practices’	• Balancing care for others and care for self • Care for others above care for self • Disguising care • Emotional support • Reciprocity • Responsibilities and obligations • Restorative • Temporary ‘windows of care’ • ‘Time out’ as self-care • Time together as ‘doing care’

### Alcohol as care for self when alone

#### ‘Time out’ as self-care

Across the sample the women described using alcohol to create a temporary respite from their roles and responsibilities of paid work, care giving and other domestic practices. They often emphasised not having to ‘think’ about their roles and responsibilities in this temporal space with alcohol. This was similar to the ‘time out’ and ‘transformative space’ described by Emslie et al. [27] ‘Time out’ with alcohol can be seen as a form of care for self because it helped the women to maintain and repair themselves, at least temporarily.

Some participants’ spent considerable time alone at home, either because they lived alone, they were single parents, or because partners were regularly out in the evenings. Thus, for these women any drinking at home would occur by and large on their own. For these women, this dynamic influenced the ways that alcohol could be an option for caring for themselves – such as a ‘time out’. For example, Diane explained that for the last 20 years she had had one drink of vodka and lemonade most evenings. She said this practice had started when her children were younger and she was alone at home most evenings, alcohol had become a way of creating ‘grown up time’ in the domestic space giving her ‘permission to relax’:.

‘.. . even before I was a single parent, [my ex-husband] worked away from home. .. Erm so the kids were in bed early on. . . I mean they would be in bed from about six,. .. I had no more interaction with them until morning. . . and I used to do it even then. .. it’s a, “This is my grown up time. This is my switch off time.”.. . I think it’s like, “This is my permission to relax” (Diane).

Therefore, alcohol could offer this ‘time out’, which was genuinely viewed as a positive form of repair, for women without adult company to drink with.

#### Care for others above care for self

Nonetheless, the accounts of Diane and the other women who were mothers, suggested that their caring responsibilities, were always a consideration for them in these practices. Most did not drink until their children went to bed, and then they ensured the quantities they drank would not stop them from being able to care for their children. For example, Penny emphasised that she did not want the drinking she did to ‘care for self’ to affect the way she looked after her children. She said *‘it’s not fair to them to wake up in a bad mood, just because you’ve had too much to drink the night before’*. While some women did drink when their children were in bed, a few of the mothers with dependent children said drinking at home alone to care for themselves when their children were there (even asleep) was not something they would do. Laura spoke about being concerned that if she drank at home she would not be able to help her children if they were ill. She said:

‘You can’t really have a good drink at home; can you, if you’ve got your kids? What happens if they take bad or something. You can’t get drunk at home.’ (Laura).

Instead Laura said she had a bath every evening to create a ‘time out’ ‘to care for herself; others had chocolate, or a cup of tea. Some also said that they had used smoking as form of care for self in the past but most had now stopped smoking. Overall, as others have found, women’s accounts of drinking practices alone emphasised being in control and moderating their drinking so they could perform their roles and responsibilities of informal care and paid work [41–43].

### Alcohol in care practices in couple relationships

#### Time together as ‘doing care’

In adult life, couple relationships are commonly seen as a key relationship where care is given and received [44]. Various writers [45, 46] have argued that in busy contemporary lives couples try to maintain ‘time together’ as a form of care. The study participants valued opportunities for care in their couple relationships, but their accounts suggested that their roles and responsibilities left limited time for this. It was notable that the participants drew on a narrative of ‘time together’ with alcohol as a way of ‘doing care’ with their partner. Jane’s example was typical, when her children were in bed on Friday nights, she and her husband drank alcohol and spent time together:

‘It’s not a drink you drink to get drunk; it’s a drink just to drink because it’s Friday, do you na what I mean? Just sit with him and spend some adult time with him. Honestly, man, it’s really important for me like to spend time with him. Ya na, after having three kids, I’ve learnt. . . we have to make time for each other.’ (Jane).

These dedicated care practices with alcohol with partners did not always involve talking or emotional support; it was simply the introduction of alcohol in these windows of time in their domestic spaces (living rooms, kitchens etc.) that marked these relationship moments as practices of care. Participants without children also spoke about drinking at home with their partner as a form of care in their relationships, away from other responsibilities such as paid work. For some participants their circumstances, in particular, child caring responsibilities or financial difficulties, meant they were limited to drinking in their home. Other women described having more freedom to drink outside the home with their partner. For example, Dawn spoke about going out to drink with her new husband once a fortnight when her children were being looked after by their father. She noted *‘We’re recently married that’s our couple time. You know so we’ll go out, we’ll go for a drink, we’ll have a meal’.* While care in couple relationships competed with other domestic practices and specific dedicated time-to-care was still often relatively infrequent, such as once a fortnight in Dawn’s account, time for care in these relationships with alcohol was seen as restorative to their couple relationships and contributing to their maintenance.

#### Balancing care for others and care for self

As others have found [47, 48] some participants were guarded about regularly drinking alone at home, however they spoke more easily about drinking at home to care for themselves when their partner was present. For example, in Penny’s account of drinking with her partner at the end of a bad day, she said that while telling her partner ‘the story’, alcohol enabled her to relax:

‘You pour it (wine) and you are telling the story and it’s going down your neck.. .. I know that that first glass quite often will go down quite quickly. .. And by the time I’ve got to the end of the story I’ve probably got to the end of the first glass.. . And the second glass is far more relaxed,. .. I use it as a tool, there’s no doubt about it, it’s a tool. And it’s probably always going to be a glass of wine.’ (Penny).

Penny described the alcohol as a ‘tool’ to repair herself and to forget about her day; drinking with her partner was more acceptable than drinking alone. Several participants spoke about routine practices of drinking with their partner at weekends, or in the evenings. In these interactions, care for self and each other, could become closely associated with routine heavy alcohol use.

A few women spoke about wanting to reduce their drinking, because the practice of drinking with their partner had become less about mutual caring and more about the consumption of alcohol itself. When participants felt that alcohol was beginning to dominate their time with their partner, this could be the trigger to feeling that they should (and often did) reduce their drinking. For example, Hannah, who said she was trying to reduce her alcohol consumption, spoke about how she used to spend most evenings drinking with her partner; they began by drinking vodka together when they got home from work, and then drank during their evening meal. Both would then continue drinking vodka while she watched television and her partner went to use his computer. She noted:

‘.. . eat dinner in front of the TV, have a few drinks erm and then he would go and play on his computer and I would stay and watch the telly. .. and drink. .. Erm – and that was - so it would be maybes like two or three hours a night. .. and we wouldn’t really be speaking to each other?. . . It’s just about, just - turning off I suppose’ (Hannah).

In Hannah’s account, the focus on caring for herself - ‘turning off’ - and the lack of elements of care that might be expected in a couple relationships - such as ‘speaking to each other’ - illustrates that regular heavy drinking, when it was all they did together, was not good enough care in this relationship. Sharon, similarly described how she and her partner had decided to reduce their drinking when they noticed how much they were consuming. She realised ‘*we weren’t doing anything else*. *. . we were sitting in’.*

### Alcohol in care practices in non-family relationships

#### Reciprocity and disguising care

The women gave many examples of care practices in non-family relationships (friends, work colleagues and neighbours); these non-family relationships enabled opportunities for receiving care that the limited time, resources, and obligations of family life sometimes did not allow for. Moreover some of these adult women did not have partners, or have family living close by. Scholars have argued that non-family relationships increasingly offer opportunities for care in adult life [49] and most of the women described that drinking with friends was an opportunity for ‘time out’ and for receiving care. For example, Kerry’s family life gave her few opportunities for receiving care or caring for herself. She also said she did not drink at home because it would affect the care she could offer her children. However, she said she was excited about going out drinking with a group of friends and the opportunity to ‘catch up:’ ‘*Saturday is just a girly night out to catch up with everybody*. *. . and it’s*. *.*. *, you know, we will have a fair bit to drink’*. Kerry described going out with friends as her ‘blow out night’. Sharon who worked full time and also carried out informal care for her grandchild and adult daughter, talked about how much she valued going for a drink with friends, who like her were mothers of adult children:

‘I think having a laugh with people is more than the alcohol. . . for helping you deal with whatever rubbish is going on. .. , just having somebody else to talk to about it makes it a hundred times better.’ (Sharon).

Sharon’s comment about *‘having a laugh.*. *. is more than the alcohol’,* suggests that going for a drink provided a socially acceptable context where reciprocal care in these relationships could occur. For other women - such as Kerry - the chemical effects of alcohol, as well as the interaction with friends, contributed to the care these practices offered them.

#### Emotional support

In contemporary life, having the opportunity to ‘talk about problems’ is considered to be indicative of emotional support and care [50]. A strong theme running through the women’s accounts was the value of ‘talking about problems’ and this was often described in drinking practices with friends. Emotional support appeared to be limited in their couple relationships in particular, and for some they did not have partners or families who they could go to for emotional support. This theme was particularly notable in two adult women who lived alone. Nina who worked long hours in a senior managerial job, described how drinking with friends on Friday night was an opportunity to talk about her problems. This helped her to care for herself at the end of the week:

‘If I’ve had a really, really bad week, by Thursday I’ll be. .. at the end of my tether, and I’ll know that on Friday. .. I know there’s a group of people who will just listen to me go, how crap my week has been. .. then I feel like I’ve sort of got rid of it and can leave it and draw a line and get ready again’ (Nina).

Nina described heavy alcohol consumption in these weekly get togethers. Overall, the overlap of drinking practices with care practices enabled opportunities for ‘repair’ that participants might not otherwise have had. The alcohol was a way of initiating these spaces or disguising their need for care.

#### Responsibilities and obligations

Drinking with non-family took place in private and public spaces. But some of the women emphasised that it was the removal and ‘time out’ from domestic spaces that also contributed to the care drinking with others offered. Drinking in public spaces with non-family enabled a ‘freedom’ from family life. This was, particularly notable for the mothers of dependent children, something captured by Andi:

‘It’s total separation from everything and just a bit… Let your hair down away from the responsibilities because you know you’re going out and you’re having a drink. Well, that’s it, you are away from it.’ (Andi).

However, for some participants the opportunities to drink in public spaces were limited. Most commonly, in addition to lack of time, this was due to child caring responsibilities, or for financial reasons. For example, one women regularly drank at her friend’s home, because it was cheaper and also because her friend’s son’s disability made it more difficult for her friend to leave the house. In all their care practices with alcohol the women always had their interdependencies and relational responsibilities to consider.

The practices with non-family sometimes involved heavy alcohol consumption, health concerns related to this were rarely mentioned or if they were, were seen as less important than the care they received. Although most acknowledged wanting to retain some control over the volume they consumed. These temporary windows of care with non-family were perceived as restorative and enabled them to carry on with their other roles and responsibilities.

### Alcohol in care practices in mother-daughter relationships

#### Care for others above care for self

Cultural expectations of motherhood present mothers as naturally caring, putting the needs of their children and partners above themselves [10, 12]. It is also assumed that mothers continue to be mothers to their children throughout their adult life [11]. Most women spoke about their own mothers at some point during the interview and many, although not all, said their mothers were an important source of care in their everyday adult life, offering both instrumental and emotional support.

It was notable that while alcohol was evident in the data in couple care practices and non-family care practices, alcohol was absent from the dyadic care practices that the women described with their mothers. They did talk about drinking with their mother at family meals and occasions, but Denise was the only women who talked about drinking alcohol with her mother in a dyadic care practice. The example she gave occurred shortly after the sudden death of Denise’s brother when Denise had moved into live with her mother, she said:

‘When I look back, it was the most horrible time of me life, I felt quite, I felt quite isolated, I felt quite lonely, me Mum was next to useless you know. .. she wasn’t particularly compassionate, it was all about her cos she’d lost a son. .. and em, and we did do a lot of heavy drinking, em to kind of get, go through the grieving process’ (Denise).

Her example of drinking with her mother is atypical in the sample, but the narrative highlights moral expectations of care from mothers, which was evident across the data. This is that mothers should put the needs of their children - including their adult children - above their own. Moreover, Denise’s account suggests that there is an element of compassion /love that is expected in mother-daughter relationships that alcohol could not offer. While Denise and her mother had drunk together and alcohol was described as providing a temporary repair - a way of forgetting about the situation - the lack of ‘compassion’ that accompanied the drinking was remarked on. Her mother’s focus on herself through drinking was seen as inappropriate, inappropriate because it was instead of offering support to her daughter, who was also in need of care. It was notable later in her interview that Denise went on to describe drinking with a new friend in a similar way to the way she had been drinking with her mother. However, in this relationship it was viewed as mutually supportive due to shared identity and reciprocity.

While no other women spoke about drinking with their mothers as a practice of care, a few spoke negatively about their mothers’ heavy drinking - if it was seen to have affected the care their mothers’ offered them.

## Discussion

Overall the findings of this study support other recent empirical work that alcohol use enables opportunities for ‘care’ in everyday relationships [26, 51]. Indeed for the women in this study, the value of care practices with alcohol with their partners and non-family members was tangible. In these relationships concerns about the health harms of alcohol were often outweighed against the openings for care alcohol provided. By using Feminist Ethics of Care to understand the findings, it can be argued that limitations in the range of care practices available to women in their personal relationships, have led to alcohol having a stronger presence in their practices of self-care. As ‘care practices’ are a necessary pre-condition for everyday wellbeing, the practices with alcohol, which are seen to do the work of ‘good care’, come to be reproduced in everyday life [18].

While there is a greater public expectation of care and emotional intimacy in couple relationships, the data supports other work that has found that private contemporary heterosexual relationships sometimes do not offer the level of care and emotional intimacy that is expected [14, 45]. Nonetheless sometimes couples come to find other ways of ‘doing care’ to demonstrate their connection [44] and drinking alcohol together may be one way of doing this. When this occurs it can solidify the importance of alcohol to dedicated caring time. However, it was noteworthy that amongst participants when drinking alcohol together with a partner became more about alcohol than caring for each other, it was viewed more negatively. It is likely that the more alcohol dominated the time with their partners, the less that time fulfilled the quality of care the women sought from their couple relationship. Thus, the expectations of the care this relationship should offer contributed to them wanting to reduce their drinking.

Other recent research has found that men use alcohol to create spaces for emotional support in non-family relationships [51, 52]. In the past some authors [53] have said that women may need alcohol less to create such space, because they can more easily and naturally gain care in their friendships. However, the qualitative data from this study challenges this gendered idea about emotional support and suggests that adult women can also have difficulty in creating non-familial reciprocal relationships. In this context alcohol can become a way of enabling care to take place. In particular, the women valued the opportunities for emotional support that drinking provided, which was absent in their other relationships. Some critiques have cautioned that while emotional support can go some way to offering care, people also need and value practical support [14, 15]. In this sample, some of the non-familial relationships had not offered care beyond the temporary emotional support offered in the drinking practices. However, other participants said that the non-family members they drank with offered support and help in other aspects of their lives, although reciprocity was important.

Cultural expectations of mothers to be nurturing and put their children’s needs above their own was a prominent theme in the data [12]. This theme was evident in the accounts of the women who were mothers, and those who were daughters. When the women who were mothers drank alcohol their accounts illustrated that they always considered their children first. The accounts of the mothers and daughters suggest that their gendered roles and relationships in families, in particular their responsibilities for care are always a consideration in their consumption of alcohol.

The few options that women had to care for self in their homes, for example smoking which is less available as such a practice now [54] could potentially contribute to a heightened use of alcohol instead. The symbolic value of alcohol and the relative ease with which it could change a domestic setting to one of care and support, including at times when alone, is likely to be contributing to its use. Some participants were reluctant to describe drinking at home alone, however many women considered alcohol to be an acceptable temporary practice of repair, if it was controlled and they had considered other people first.

Overall, the women’s accounts of alcohol use within care practices both with others and alone can be linked to unequal gender relations and their reproduction. Alcohol aided the creation of small temporary windows of care in their couple relationships, and non-family relationships. However, because all it did was help the women feel better temporarily, it did little to mitigate against the inequalities embedded in their care responsibilities and other life challenges [30]. When the care practices with alcohol interfered with their functioning in other aspects of life, such as the gendered responsibility of looking after their family or being able to carry out their job, it came to be viewed by the women themselves, as well as by others, as less acceptable. Overall, the data suggests that alcohol is increasingly being used in practices of care in many relationships, fulfilling women’s’ needs for care, while increasing heavy drinking.

### Limitations

A limitation of this study is the relatively small sample size which cannot be extrapolated to the wider populations. There has only been limited room to discuss how care practices with alcohol might be reproduced differently in women with different social and economic positions, but certainly within the sample some had more options for alternative practices than others [55]. These intersectional differences require further exploration.

## Conclusions

The study supports others [54, 55] who argue for the potential of a social practices approach for making inferences about intervention approaches. Rather than viewing lifestyle behaviours such as alcohol use, smoking, eating, as standalone behaviours that can be changed at an individual level and placing responsibility on individuals to make changes to themselves only [56], a social practices approach sees these behaviours as embedded within practices of everyday life [54, 56]. By viewing alcohol within care practices, when thinking of approaches to intervention around women’s alcohol use, the value of care and relationships need to be acknowledged. This study has important practical implications, complementing the growing body of work that is highlighting relational interventions [16, 17]. It suggests that widening the limited care networks, and promoting the importance of care practices for self, alongside care for others could be beneficial. There are possibilities for working with women to enable women’s support networks, to offer and ask for care outside relationships where it is normally expected, and also help them to see the reasons for inequalities in their lives [57, 58]. These approaches might help to address the finding that for some women, alcohol was one of the only forms of care available.

## Additional file

Additional file 1: This file is the study interview schedule. The questions in the schedule were used to guide the discussions during the semi-structured interviews. (DOCX 21 kb)
