# Supplementary material for: Understanding alcohol as an element of ‘care practices’ in adult White British women’s everyday personal relationships: a qualitative study
Source: BMC Womens Health. 2018 Sep 5;18:137. doi: 10.1186/s12905-018-0629-6 (PMC6123968; doi:10.1186/s12905-018-0629-6)
Supplement: Supplementary file 1 — This file is the study interview schedule. The questions in the schedule were used to guide the discussions during the semi-structured interviews. (DOCX 21 kb) [file 12905_2018_629_MOESM1_ESM.docx]

**Additional File 1 - Study Interview Schedule**

**Everyday life and stress**

Please could you begin by telling me a bit about your everyday life at the moment? What does a typical week involve for you? *Probe: home/ work/ leisure/ family*

Can you tell me about the main sources of stress in your life at the moment? *Probe: recent examples - home/ work*

What kind of things help you to manage times of stress? *Probe: social support/ health behaviours/ leisure/ hobbies, religion etc.*

What things are you more likely to do when you are having a particularly stressful day? On the other hand, are there things you are less likely to do?

**Stress and alcohol use**

Can you describe an occasion when you might drink alcohol because you are feeling stressed in your day to day life at the moment? *Probe: where, when, what, who with, how much*, *explore how this might fit with other health behaviours e.g. smoking*

What is it about alcohol in that situation?

What is different about the times you drink alcohol when you are feeling stressed and times you don’t?

How do the stresses and strains you experience now compare to those you’ve experienced at other times in your life? *Probe: what they have been at other times and particular pressures points, transitions*

Could you tell me about your alcohol use at these points in your life? *Probe: how much, where, who with*

Please could tell me about how drinking when experiencing stress makes you feel? *Probe: better, worse, emotionally/physically*

**Alcohol use**

I’d like to talk about your alcohol use more generally - What would be a typical day/time when you drink alcohol? *Probe: where, when, what, who with, how much*

Can you tell me a bit about your drinking throughout your life, how does your drinking now compare to drinking at other times in your life?

Are there times when your alcohol use has been heavier*?* Are there times when your drinking has been lighter? *Probe: experiences of heavier/ lighter times and reasons*

Could you tell me about your drinking when you are by yourself / compared to when you are with other people?

Could you tell me about your families drinking as you grew up, and now? *Probe: parents, siblings, partner* etc.

How would you describe other people who are important in your life’s’ views about drinking? *Probe: family, friends, neighbours*

How would you describe the drinking ‘culture’ in your work place? *Probe: examples of colleagues’ behaviour*

How would you describe problematic drinking? Where might you consider someone’s drinking to be a problem?

Have you ever been concerned about your own drinking or tried to cut back? *Probe: what they did?*

**Closing questions**

Is there anything else you wanted to say about your alcohol and / or stress that we haven’t talked about?

**Thank and close**
